# 5-Acetyl-2-amino-4-(2-fluoro­phen­yl)-6-methyl-4*H*-pyran-3-carbo­nitrile dichlo­methane hemisolvate

**DOI:** 10.1107/S2414314625003372

**Published:** 2025-04-24

**Authors:** Carren Nyapola, Sizwe J. Zamisa, Bernard Omondi, Eric M. Njogu

**Affiliations:** a University of KwaZulu-Natal, School of Chemistry and Physics, Private bag X54001, Durban, 4000, South Africa; b Multimedia University of Kenya, PO Box 15653-00503, Nairobi, Kenya; University of Aberdeen, United Kingdom

**Keywords:** crystal structure, pyran, hydrogen bond

## Abstract

The title compound, 2C_15_H_13_FN_2_O_2_·CH_2_Cl_2_, crystallizes with two main mol­ecules in the asymmetric unit with a disordered di­chloro­methane solvent mol­ecule. The dihedral angles between the fluoro phenyl ring and 4*H*-pyran ring are 74.36 (15) and 80.69 (15)° in the two mol­ecules. In the crystal, N—H⋯N and N—H⋯O hydrogen bonds link the mol­ecules into a two-dimensional supra­molecular network propagating in the (100) plane.

## Abstract 2

A two-dimensional supra­molecular architecture mediated by N—H⋯N and N—H⋯O hydrogen bonds is formed in the crystal of the title compound.

## Graphical abstract

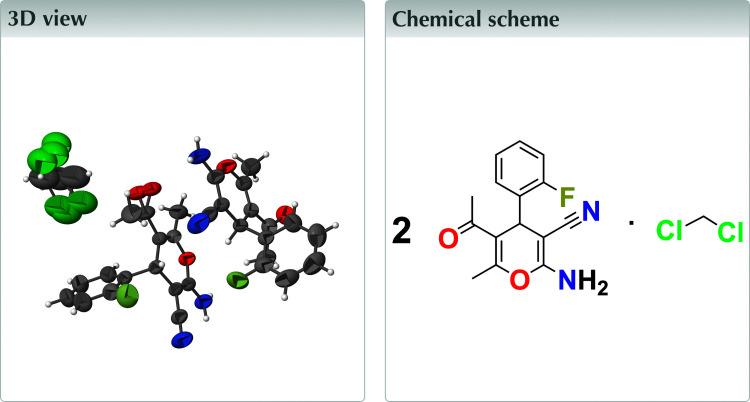

## Structure description

Amino-4*H*-pyran derivatives are useful building blocks in creating pharmacologically active heterocycles in multicomponent reactions, such as anti-tumor (Fouda, 2016 ▸), anti­bacterial (Kathrotiya & Patel, 2012 ▸), anti­mycobacterial (Alvey *et al.*, 2009 ▸) anti­leishmanial (Narender & Gupta, 2004 ▸) and anti­proliferative agents (Mansouri *et al.*, 2011 ▸). Our previous study on aryl-based 2-amino carbo­nitriles identified different hydrogen-bonding patters in the crystal structures (Zamisa *et al.*, 2022 ▸). A structural analysis using the Cambridge Structural Database (CSD version 5.46, November 2024 update); Groom *et al.*, 2016 ▸) showed various types of hydrogen-bonding patterns driven by substituents on the 4*H*-pyran core. These were dominated by N—H⋯N and N—H⋯O inter­actions involving the amino and carbon­yl/cyano groups. This analysis indicated that the binding affinity of these compounds towards calf thymus de­oxy­ribonucleic acid may be associated with hydrogen bonds involving their amino functional groups. This finding is consistent with our exploration of potential anti­cancer agents (Zamisa *et al.*, 2022 ▸). The current study continues our investigation into the structures of 4*H*-pyran derivatives as potential anti­cancer agents and reports the synthesis and structure of the title compound.

**Figure 1:**
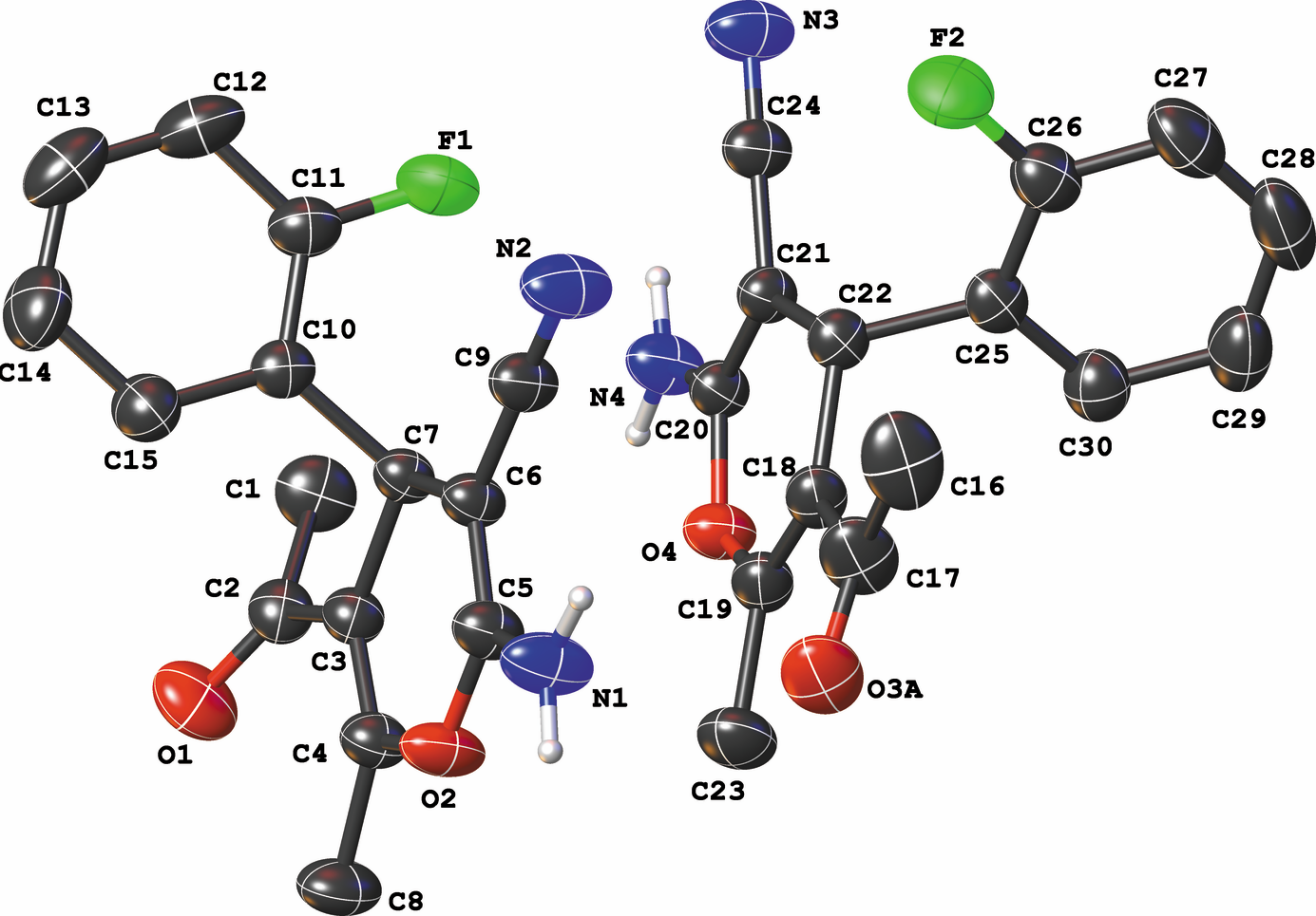
The mol­ecular structure of the title compound with displacement ellipsoids drawn at the 40% probability level. The disordered components, including the aromatic and methine hydrogen atoms, are omitted for clarity.

There are two symmetrically independent C_15_H_13_FN_2_O_2_ mol­ecules in the asymmetric unit (Fig. 1 ▸). Each mol­ecule comprises a cyclo­alkanone, 4*H*-pyran core with attached phenyl moiety, cyano, and amino groups. The dihedral angle between the fluoro­phenyl and 4*H*-pyran rings are 74.36 (15)° (C1 mol­ecule) and 80.69 (15)° (C16 mol­ecule) and are similar to those of related compounds in the literature (Zamisa *et al.*, 2022 ▸, 2023 ▸).

**Figure 2:**
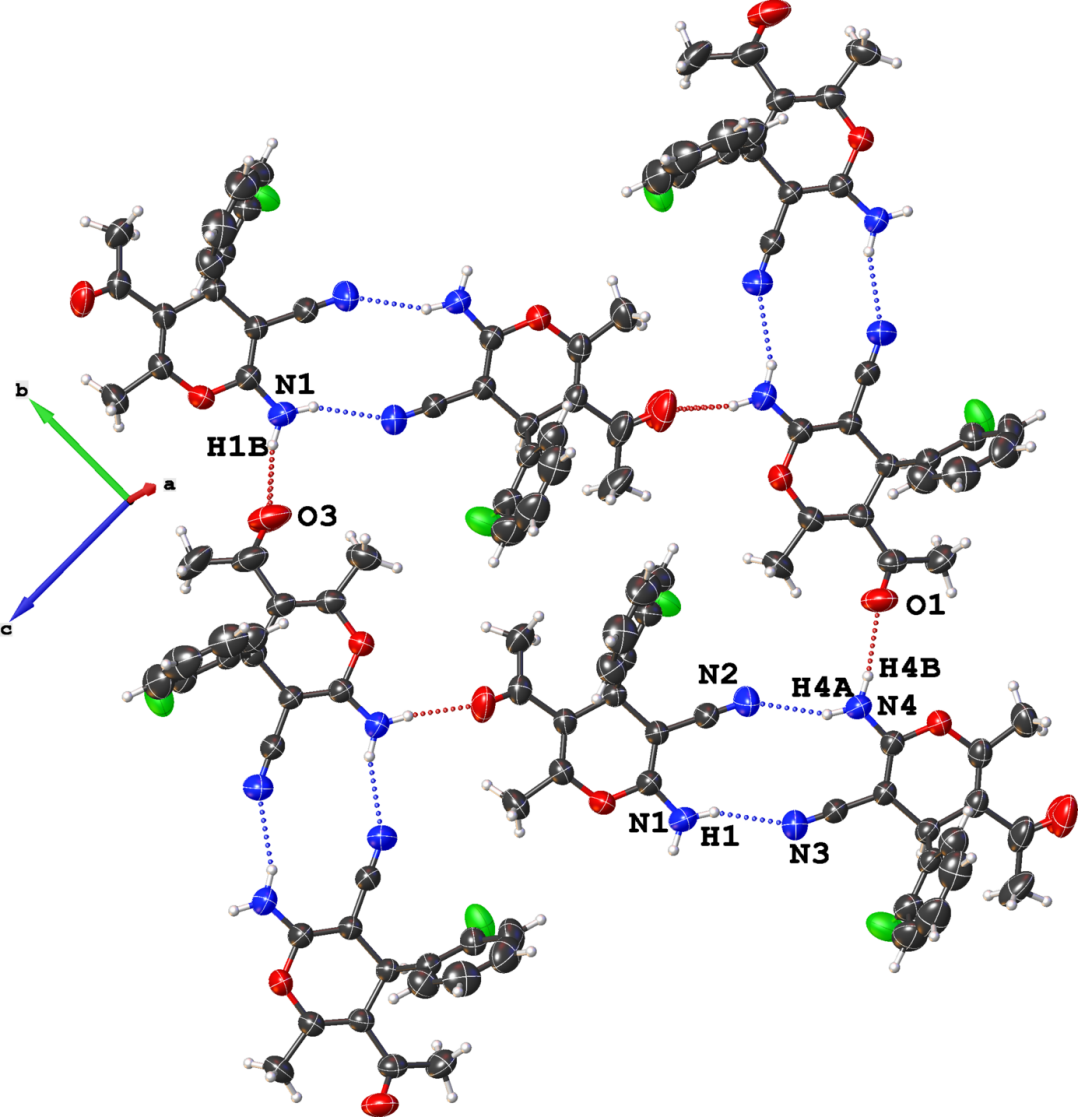
A projection of the crystal packing along the *a*-axis. Dashed lines denote N—H⋯N and N—H⋯O hydrogen bonds. Motifs **1** and **II** (see text) are depicted by blue and red dotted lines, respectively.

In the crystal, the mol­ecules are linked by N—H⋯N and N—H⋯O hydrogen bonds (Table 1 ▸, Fig. 2 ▸) engendered by the amine functional group. One of these hydrogen atoms, H1*B* or H4*B*, inter­acts with the nitro­gen atom N3 or N2 of a neighbouring mol­ecule *via* an N—H⋯N link with the graph-set descriptor 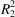
(12) (involving the amino and the cyano group) (Motif **I**). An N—H⋯O hydrogen bond graph with graph-set descriptor 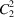
(10) with the carbonyl group oxygen atom O3 or O1 of a neighbouring mol­ecule acting as acceptor to the amine (Motif **II**), Fig. 2 ▸ (Nyapola *et al.*, 2024 ▸; Zamisa *et al.*, 2022 ▸) is also observed. These two motifs combined create a supra­molecular structure that propagates in the (100) plane of the crystal.

## Synthesis and crystallization

0.015 mmol of 1,3-cyclo­hexa­nedione were mixed with 0.015 mmol of malonotrile and 0.015 mmol of benzaldehyde in a microwave vessel. A catalytic amount of tri­ethyl­amine was added in a tightly sealed 35 ml microwave reaction vessel, and the mixture was subjected to microwave radiation at 150°C for 10 minutes. An off-white solid precipitate was formed and collected by vacuum filtration. The reaction progress was monitored using thin-layer chromatography with a solvent ratio of 1:1 for ethyl acetate and hexane. The resulting precipitate was isolated and recrystallized from ethanol solution. Crystals of the title compound were obtained through slow isothermal evaporation from absolute di­chloro­methane solution.

## Refinement

Crystallographic data and structure refinement details are summarized in Table 2 ▸. SIMU restraints and EADP constraints in *SHELXL* were used to model the disorder of the solvent mol­ecule. The hydrogen atoms were positioned geometrically with N—H = 0.86 Å and C—H = 0.93–0.96 Å depending on hybridization and allowed to ride on their parent atoms with *U*_iso_(H) = 1.2*U*_eq_(C).

## Supplementary Material

Crystal structure: contains datablock(s) I. DOI: 10.1107/S2414314625003372/hb4509sup1.cif

Structure factors: contains datablock(s) I. DOI: 10.1107/S2414314625003372/hb4509Isup2.hkl

Supporting information file. DOI: 10.1107/S2414314625003372/hb4509Isup3.cml

CCDC reference: 2443758

Additional supporting information:  crystallographic information; 3D view; checkCIF report

Additional supporting information:  crystallographic information; 3D view; checkCIF report

## Figures and Tables

**Table 1:** Hydrogen-bond geometry (Å, °)

*D*—H⋯*A*	*D*—H	H⋯*A*	*D*⋯*A*	*D*—H⋯*A*
N1—H1*A*⋯N3^i^	0.86	2.26	3.066 (4)	156
N1—H1*B*⋯O3^ii^	0.86	2.03	2.844 (8)	159
N1—H1*B*⋯O3*A*^ii^	0.86	2.02	2.855 (7)	165
N4—H4*A*⋯N2^iii^	0.86	2.20	3.016 (4)	157
N4—H4*B*⋯O1^iv^	0.86	2.06	2.910 (3)	170

Symmetry codes: (i) 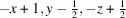; (ii) 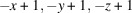; (iii) 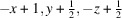; (iv) 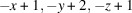.

**Table 2:** Experimental details

Crystal data	
Chemical formula	2C_15_H_13_FN_2_O_2_·CH_2_Cl_2_
*M*_r_	629.47
Crystal system, space group	Monoclinic, *P*2_1_/*c*
Temperature (K)	296
*a*, *b*, *c* (Å)	10.8670 (4), 15.2671 (6), 19.1569 (8)
β (°)	97.463 (2)
*V* (Å^3^)	3151.4 (2)
*Z*	4
Radiation type	Mo *K*α
μ (mm^−1^)	0.26
Crystal size (mm)	0.31 × 0.22 × 0.16

Data collection	
Diffractometer	Bruker *SMART* APEXII CCD
Absorption correction	Multi-scan (*SADABS*; Krause *et al.*, 2015 ▸)
*T*_min_, *T*_max_	0.485, 0.746
No. of measured, independent and observed [*I* > 2σ(*I*)] reflections	84575, 6190, 4405
*R*_int_	0.063
(sin θ/λ)_max_ (Å^−1^)	0.617

Refinement	
*R*[*F*^2^ > 2σ(*F*^2^)], *wR*(*F*^2^), *S*	0.074, 0.239, 1.08
No. of reflections	6190
No. of parameters	419
No. of restraints	59
H-atom treatment	H-atom parameters constrained
Δρ_max_, Δρ_min_ (e Å^−3^)	0.71, −0.61

Computer programs: *APEX2* and *SAINT* (Bruker, 2016 ▸), *SHELXT2018/2* (Sheldrick, 2015*a* ▸), *SHELXL2018/3* (Sheldrick, 2015*b* ▸) and *OLEX2* 1.5 (Dolomanov *et al.*, 2009 ▸).

## full crystallographic data

### 5-Acetyl-2-amino-4-(2-fluorophenyl)-6-methyl-4H-pyran-3-carbonitrile dichloromethane hemisolvate Crystal data

**Table:**

2C_15_H_13_FN_2_O_2_·CH_2_Cl_2_	*F*(000) = 1304
*M**_r_* = 629.47	*D*_x_ = 1.327 Mg m^−^^3^
Monoclinic, *P*2_1_/*c*	Mo *K*α radiation, λ = 0.71073 Å
*a* = 10.8670 (4) Å	Cell parameters from 9748 reflections
*b* = 15.2671 (6) Å	θ = 2.3–27.0°
*c* = 19.1569 (8) Å	µ = 0.26 mm^−^^1^
β = 97.463 (2)°	*T* = 296 K
*V* = 3151.4 (2) Å^3^	Block, colourless
*Z* = 4	0.31 × 0.22 × 0.16 mm

### 5-Acetyl-2-amino-4-(2-fluorophenyl)-6-methyl-4H-pyran-3-carbonitrile dichloromethane hemisolvate Data collection

**Table:**

Bruker SMART APEXII CCD diffractometer	6190 independent reflections
Radiation source: microfocus sealed X-ray tube, Incoatec Iµs	4405 reflections with *I* > 2σ(*I*)
Mirror optics monochromator	*R*_int_ = 0.063
Detector resolution: 7.9 pixels mm^-1^	θ_max_ = 26.0°, θ_min_ = 1.7°
ω and φ scans	*h* = −13→13
Absorption correction: multi-scan (SADABS; Krause *et al.*, 2015)	*k* = −18→18
*T*_min_ = 0.485, *T*_max_ = 0.746	*l* = −23→23
84575 measured reflections	

### 5-Acetyl-2-amino-4-(2-fluorophenyl)-6-methyl-4H-pyran-3-carbonitrile dichloromethane hemisolvate Refinement

**Table:**

Refinement on *F*^2^	Primary atom site location: dual
Least-squares matrix: full	Hydrogen site location: inferred from neighbouring sites
*R*[*F*^2^ > 2σ(*F*^2^)] = 0.074	H-atom parameters constrained
*wR*(*F*^2^) = 0.239	*w* = 1/[σ^2^(*F*_o_^2^) + (0.1098*P*)^2^ + 1.8679*P*] where *P* = (*F*_o_^2^ + 2*F*_c_^2^)/3
*S* = 1.08	(Δ/σ)_max_ < 0.001
6190 reflections	Δρ_max_ = 0.71 e Å^−^^3^
419 parameters	Δρ_min_ = −0.60 e Å^−^^3^
59 restraints	

### 5-Acetyl-2-amino-4-(2-fluorophenyl)-6-methyl-4H-pyran-3-carbonitrile dichloromethane hemisolvate Special details

**Table:**

Geometry. All esds (except the esd in the dihedral angle between two l.s. planes) are estimated using the full covariance matrix. The cell esds are taken into account individually in the estimation of esds in distances, angles and torsion angles; correlations between esds in cell parameters are only used when they are defined by crystal symmetry. An approximate (isotropic) treatment of cell esds is used for estimating esds involving l.s. planes.

### 5-Acetyl-2-amino-4-(2-fluorophenyl)-6-methyl-4H-pyran-3-carbonitrile dichloromethane hemisolvate Fractional atomic coordinates and isotropic or equivalent isotropic displacement parameters (Å^2^)

**Table:**

	*x*	*y*	*z*	*U*_iso_*/*U*_eq_	Occ. (<1)
F1	0.6616 (2)	0.83522 (16)	0.26782 (10)	0.0953 (7)	
O1	0.7001 (3)	0.93283 (16)	0.55386 (12)	0.0889 (7)	
O2	0.6515 (2)	0.66060 (13)	0.52455 (10)	0.0754 (6)	
N1	0.6603 (3)	0.53973 (16)	0.46177 (14)	0.0874 (9)	
H1A	0.664961	0.509947	0.424097	0.105*	
H1B	0.655578	0.513218	0.500938	0.105*	
N2	0.6820 (4)	0.60700 (19)	0.28440 (15)	0.0938 (10)	
C1	0.6117 (3)	0.9631 (2)	0.43838 (18)	0.0780 (9)	
H1C	0.672777	0.970770	0.406908	0.117*	
H1D	0.592906	1.018817	0.457700	0.117*	
H1E	0.537620	0.938604	0.413090	0.117*	
C2	0.6612 (3)	0.90264 (18)	0.49666 (15)	0.0590 (7)	
C3	0.6610 (2)	0.80710 (17)	0.48275 (13)	0.0512 (6)	
C4	0.6532 (3)	0.74986 (18)	0.53509 (14)	0.0592 (7)	
C5	0.6604 (3)	0.62692 (18)	0.45944 (14)	0.0616 (7)	
C6	0.6671 (3)	0.67926 (16)	0.40317 (13)	0.0524 (6)	
C7	0.6665 (2)	0.77841 (15)	0.40747 (13)	0.0487 (6)	
H7	0.591720	0.799862	0.378372	0.058*	
C8	0.6404 (4)	0.7660 (2)	0.61059 (16)	0.0832 (10)	
H8A	0.720099	0.760602	0.638425	0.125*	
H8B	0.584349	0.723771	0.626143	0.125*	
H8C	0.608424	0.823891	0.615798	0.125*	
C9	0.6762 (3)	0.63938 (18)	0.33777 (15)	0.0617 (7)	
C10	0.7795 (2)	0.81656 (16)	0.37852 (13)	0.0518 (6)	
C11	0.7726 (3)	0.84350 (19)	0.30958 (16)	0.0651 (7)	
C12	0.8707 (4)	0.8794 (2)	0.2811 (2)	0.0873 (11)	
H12	0.861562	0.896505	0.234132	0.105*	
C13	0.9811 (4)	0.8896 (3)	0.3224 (3)	0.0961 (12)	
H13	1.048277	0.913937	0.303786	0.115*	
C14	0.9936 (3)	0.8638 (3)	0.3919 (2)	0.0920 (11)	
H14	1.068962	0.871236	0.420324	0.110*	
C15	0.8935 (3)	0.8266 (2)	0.41938 (18)	0.0727 (8)	
H15	0.903207	0.808215	0.466051	0.087*	
F2	0.3253 (2)	0.65517 (16)	0.13286 (11)	0.1017 (7)	
O4	0.30673 (19)	0.84069 (12)	0.38524 (9)	0.0618 (5)	
N3	0.3590 (3)	0.89205 (18)	0.14758 (15)	0.0865 (9)	
N4	0.3104 (3)	0.96089 (15)	0.32094 (13)	0.0688 (7)	
H4A	0.316273	0.990322	0.283234	0.083*	
H4B	0.300445	0.987702	0.359285	0.083*	
C16	0.3714 (4)	0.5364 (2)	0.3085 (2)	0.0996 (13)	
H16A	0.297518	0.526520	0.276201	0.149*	
H16B	0.399092	0.482090	0.330364	0.149*	
H16C	0.435043	0.559851	0.283542	0.149*	
C17	0.3444 (4)	0.5994 (2)	0.3631 (2)	0.0860 (10)	
C18	0.3294 (2)	0.69418 (17)	0.34666 (15)	0.0562 (6)	
C19	0.3188 (3)	0.75174 (18)	0.39809 (15)	0.0584 (7)	
C20	0.3169 (2)	0.87347 (16)	0.31995 (13)	0.0516 (6)	
C21	0.3321 (2)	0.82051 (16)	0.26544 (13)	0.0497 (6)	
C22	0.3258 (2)	0.72140 (15)	0.27028 (14)	0.0516 (6)	
H22	0.398628	0.696739	0.252122	0.062*	
C23	0.3164 (4)	0.7376 (3)	0.47495 (17)	0.0837 (10)	
H23A	0.238184	0.712484	0.482371	0.100*	
H23B	0.327234	0.792666	0.499163	0.100*	
H23C	0.382327	0.698536	0.492790	0.100*	
C24	0.3476 (3)	0.85970 (17)	0.20039 (15)	0.0597 (7)	
C25	0.2090 (3)	0.68716 (16)	0.22507 (14)	0.0550 (6)	
C26	0.2138 (3)	0.6560 (2)	0.15778 (17)	0.0715 (8)	
C27	0.1119 (5)	0.6249 (3)	0.1150 (2)	0.0957 (12)	
H27	0.119165	0.604588	0.069968	0.115*	
C28	−0.0002 (4)	0.6245 (3)	0.1398 (3)	0.1032 (14)	
H28	−0.069957	0.603421	0.111521	0.124*	
C29	−0.0109 (3)	0.6550 (3)	0.2067 (2)	0.0913 (11)	
H29	−0.087412	0.654609	0.223550	0.110*	
C30	0.0939 (3)	0.6862 (2)	0.24847 (18)	0.0711 (8)	
H30	0.086499	0.707098	0.293346	0.085*	
O3A	0.3692 (6)	0.5749 (4)	0.4238 (4)	0.0913 (13)	0.5
O3	0.2981 (7)	0.5700 (4)	0.4175 (4)	0.107 (2)	0.5
Cl2	−0.0289 (13)	0.5107 (10)	0.3527 (10)	0.183 (2)	0.3333
Cl3	−0.0044 (12)	0.3416 (6)	0.4020 (5)	0.1845 (17)	0.3333
C1A	0.0493 (19)	0.4488 (8)	0.4202 (10)	0.182 (2)	0.3333
H1AA	0.029605	0.468328	0.465648	0.219*	0.3333
H1AB	0.138398	0.452374	0.419922	0.219*	0.3333
Cl3A	0.0105 (8)	0.3565 (4)	0.4722 (4)	0.1845 (17)	0.3333
C1B	−0.045 (2)	0.3910 (15)	0.3879 (10)	0.182 (2)	0.3333
H1BA	0.002048	0.358295	0.356847	0.219*	0.3333
H1BB	−0.129373	0.369594	0.378881	0.219*	0.3333
Cl2A	−0.0494 (14)	0.4896 (11)	0.3592 (10)	0.183 (2)	0.3333
Cl1	0.0398 (7)	0.5174 (6)	0.3831 (4)	0.183 (2)	0.3333
Cl4	0.0405 (12)	0.3494 (5)	0.4256 (5)	0.1845 (17)	0.3333
C1C	−0.0492 (18)	0.4449 (9)	0.4279 (13)	0.182 (2)	0.3333
H1CA	−0.054082	0.464065	0.475786	0.219*	0.3333
H1CB	−0.132263	0.437309	0.403269	0.219*	0.3333

### 5-Acetyl-2-amino-4-(2-fluorophenyl)-6-methyl-4H-pyran-3-carbonitrile dichloromethane hemisolvate Atomic displacement parameters (Å^2^)

**Table:**

	*U*^11^	*U*^22^	*U*^33^	*U*^12^	*U*^13^	*U*^23^
F1	0.1047 (15)	0.1224 (18)	0.0571 (11)	−0.0045 (12)	0.0040 (10)	0.0216 (10)
O1	0.123 (2)	0.0682 (14)	0.0725 (15)	0.0101 (13)	0.0004 (13)	−0.0274 (11)
O2	0.1291 (19)	0.0538 (11)	0.0446 (10)	−0.0029 (11)	0.0166 (11)	0.0041 (8)
N1	0.156 (3)	0.0467 (14)	0.0599 (15)	−0.0021 (15)	0.0174 (16)	0.0057 (11)
N2	0.159 (3)	0.0625 (16)	0.0641 (17)	−0.0180 (18)	0.0316 (18)	−0.0145 (14)
C1	0.098 (2)	0.0503 (16)	0.086 (2)	0.0146 (15)	0.0117 (18)	−0.0010 (15)
C2	0.0612 (15)	0.0557 (15)	0.0615 (17)	0.0043 (12)	0.0134 (13)	−0.0107 (13)
C3	0.0566 (14)	0.0501 (14)	0.0474 (13)	0.0034 (11)	0.0088 (11)	−0.0045 (11)
C4	0.0758 (18)	0.0559 (15)	0.0466 (14)	0.0005 (13)	0.0101 (12)	−0.0029 (11)
C5	0.087 (2)	0.0474 (14)	0.0506 (15)	0.0006 (13)	0.0084 (13)	0.0000 (11)
C6	0.0701 (16)	0.0441 (13)	0.0430 (13)	−0.0012 (11)	0.0075 (11)	−0.0013 (10)
C7	0.0588 (14)	0.0431 (13)	0.0435 (13)	0.0024 (10)	0.0046 (10)	0.0008 (10)
C8	0.121 (3)	0.083 (2)	0.0489 (17)	−0.007 (2)	0.0238 (17)	−0.0078 (15)
C9	0.087 (2)	0.0458 (14)	0.0530 (16)	−0.0081 (13)	0.0119 (13)	−0.0026 (12)
C10	0.0630 (15)	0.0409 (12)	0.0526 (14)	0.0041 (11)	0.0120 (11)	−0.0025 (10)
C11	0.0790 (19)	0.0589 (16)	0.0594 (17)	0.0044 (14)	0.0160 (14)	0.0053 (13)
C12	0.104 (3)	0.081 (2)	0.085 (2)	0.010 (2)	0.045 (2)	0.0221 (19)
C13	0.091 (3)	0.082 (2)	0.126 (4)	−0.004 (2)	0.055 (3)	0.006 (2)
C14	0.0598 (19)	0.107 (3)	0.112 (3)	−0.0048 (18)	0.0196 (19)	−0.012 (2)
C15	0.0657 (18)	0.084 (2)	0.0692 (19)	0.0023 (15)	0.0119 (14)	−0.0028 (16)
F2	0.1180 (17)	0.1154 (18)	0.0760 (13)	−0.0059 (13)	0.0281 (12)	−0.0268 (12)
O4	0.0872 (13)	0.0507 (10)	0.0483 (10)	0.0004 (9)	0.0120 (9)	0.0018 (8)
N3	0.138 (3)	0.0617 (16)	0.0629 (17)	−0.0072 (16)	0.0236 (16)	0.0076 (13)
N4	0.109 (2)	0.0436 (12)	0.0536 (13)	−0.0007 (12)	0.0116 (13)	−0.0040 (10)
C16	0.113 (3)	0.0431 (17)	0.135 (3)	0.0125 (17)	−0.012 (2)	0.0041 (19)
C17	0.110 (2)	0.0546 (16)	0.088 (2)	−0.0015 (16)	−0.0097 (19)	0.0247 (15)
C18	0.0607 (15)	0.0455 (13)	0.0601 (16)	−0.0011 (11)	−0.0005 (12)	0.0102 (12)
C19	0.0614 (15)	0.0557 (15)	0.0562 (16)	−0.0037 (12)	0.0001 (12)	0.0113 (12)
C20	0.0607 (15)	0.0443 (13)	0.0492 (14)	−0.0031 (11)	0.0049 (11)	0.0016 (10)
C21	0.0600 (14)	0.0405 (12)	0.0487 (13)	−0.0032 (10)	0.0073 (11)	0.0009 (10)
C22	0.0581 (14)	0.0394 (12)	0.0570 (15)	0.0029 (10)	0.0063 (11)	0.0013 (10)
C23	0.105 (3)	0.087 (2)	0.0570 (18)	−0.0057 (19)	0.0022 (17)	0.0175 (16)
C24	0.0834 (19)	0.0422 (13)	0.0542 (16)	−0.0032 (12)	0.0119 (13)	−0.0025 (12)
C25	0.0666 (16)	0.0366 (12)	0.0602 (16)	0.0000 (11)	0.0021 (12)	0.0018 (11)
C26	0.090 (2)	0.0567 (17)	0.0664 (19)	−0.0031 (15)	0.0030 (16)	−0.0045 (14)
C27	0.125 (3)	0.083 (2)	0.072 (2)	−0.013 (2)	−0.016 (2)	−0.0143 (18)
C28	0.106 (3)	0.087 (3)	0.103 (3)	−0.025 (2)	−0.040 (3)	0.007 (2)
C29	0.067 (2)	0.094 (3)	0.108 (3)	−0.0108 (18)	−0.0091 (19)	0.015 (2)
C30	0.0692 (19)	0.0662 (18)	0.076 (2)	−0.0005 (15)	0.0018 (15)	0.0026 (15)
O3A	0.116 (3)	0.060 (2)	0.092 (2)	−0.001 (2)	−0.008 (2)	0.0293 (18)
O3	0.132 (5)	0.073 (3)	0.108 (4)	−0.012 (4)	−0.008 (4)	0.044 (3)
Cl2	0.183 (5)	0.198 (5)	0.172 (4)	0.039 (4)	0.029 (3)	0.042 (3)
Cl3	0.249 (6)	0.148 (2)	0.152 (5)	0.028 (3)	0.007 (4)	−0.019 (3)
C1A	0.181 (5)	0.196 (5)	0.171 (4)	0.040 (4)	0.026 (4)	0.044 (4)
Cl3A	0.249 (6)	0.148 (2)	0.152 (5)	0.028 (3)	0.007 (4)	−0.019 (3)
C1B	0.181 (5)	0.196 (5)	0.171 (4)	0.040 (4)	0.026 (4)	0.044 (4)
Cl2A	0.183 (5)	0.198 (5)	0.172 (4)	0.039 (4)	0.029 (3)	0.042 (3)
Cl1	0.183 (5)	0.198 (5)	0.172 (4)	0.039 (4)	0.029 (3)	0.042 (3)
Cl4	0.249 (6)	0.148 (2)	0.152 (5)	0.028 (3)	0.007 (4)	−0.019 (3)
C1C	0.181 (5)	0.196 (5)	0.171 (4)	0.040 (4)	0.026 (4)	0.044 (4)

### 5-Acetyl-2-amino-4-(2-fluorophenyl)-6-methyl-4H-pyran-3-carbonitrile dichloromethane hemisolvate Geometric parameters (Å, º)

**Table:**

F1—C11	1.364 (4)	C16—H16A	0.9600
O1—C2	1.213 (3)	C16—H16B	0.9600
O2—C4	1.377 (3)	C16—H16C	0.9600
O2—C5	1.364 (3)	C16—C17	1.477 (6)
N1—H1A	0.8600	C17—C18	1.486 (4)
N1—H1B	0.8600	C17—O3A	1.217 (7)
N1—C5	1.332 (4)	C17—O3	1.293 (8)
N2—C9	1.145 (4)	C18—C19	1.336 (4)
C1—H1C	0.9600	C18—C22	1.517 (4)
C1—H1D	0.9600	C19—C23	1.492 (4)
C1—H1E	0.9600	C20—C21	1.348 (4)
C1—C2	1.494 (4)	C21—C22	1.518 (3)
C2—C3	1.483 (4)	C21—C24	1.412 (4)
C3—C4	1.341 (4)	C22—H22	0.9800
C3—C7	1.516 (3)	C22—C25	1.533 (4)
C4—C8	1.491 (4)	C23—H23A	0.9600
C5—C6	1.351 (4)	C23—H23B	0.9600
C6—C7	1.516 (3)	C23—H23C	0.9600
C6—C9	1.408 (4)	C25—C26	1.381 (4)
C7—H7	0.9800	C25—C30	1.382 (4)
C7—C10	1.527 (4)	C26—C27	1.374 (5)
C8—H8A	0.9600	C27—H27	0.9300
C8—H8B	0.9600	C27—C28	1.363 (6)
C8—H8C	0.9600	C28—H28	0.9300
C10—C11	1.376 (4)	C28—C29	1.383 (6)
C10—C15	1.385 (4)	C29—H29	0.9300
C11—C12	1.372 (5)	C29—C30	1.389 (5)
C12—H12	0.9300	C30—H30	0.9300
C12—C13	1.358 (6)	Cl2—C1A	1.734 (8)
C13—H13	0.9300	Cl3—C1A	1.757 (8)
C13—C14	1.377 (6)	C1A—H1AA	0.9700
C14—H14	0.9300	C1A—H1AB	0.9700
C14—C15	1.390 (5)	Cl3A—C1B	1.730 (16)
C15—H15	0.9300	C1B—H1BA	0.9700
F2—C26	1.359 (4)	C1B—H1BB	0.9700
O4—C19	1.383 (3)	C1B—Cl2A	1.601 (18)
O4—C20	1.365 (3)	Cl1—C1C	1.765 (9)
N3—C24	1.147 (4)	Cl4—C1C	1.758 (9)
N4—H4A	0.8600	C1C—H1CA	0.9700
N4—H4B	0.8600	C1C—H1CB	0.9700
N4—C20	1.337 (3)		
			
C5—O2—C4	120.4 (2)	C16—C17—C18	120.6 (3)
H1A—N1—H1B	120.0	O3A—C17—C16	115.9 (5)
C5—N1—H1A	120.0	O3A—C17—C18	120.4 (5)
C5—N1—H1B	120.0	O3—C17—C16	118.6 (4)
H1C—C1—H1D	109.5	O3—C17—C18	117.7 (5)
H1C—C1—H1E	109.5	C17—C18—C22	117.4 (3)
H1D—C1—H1E	109.5	C19—C18—C17	120.1 (3)
C2—C1—H1C	109.5	C19—C18—C22	122.5 (2)
C2—C1—H1D	109.5	O4—C19—C23	107.7 (3)
C2—C1—H1E	109.5	C18—C19—O4	122.0 (2)
O1—C2—C1	119.3 (3)	C18—C19—C23	130.3 (3)
O1—C2—C3	121.9 (3)	N4—C20—O4	110.0 (2)
C3—C2—C1	118.8 (2)	N4—C20—C21	128.5 (2)
C2—C3—C7	117.1 (2)	C21—C20—O4	121.5 (2)
C4—C3—C2	120.4 (2)	C20—C21—C22	122.7 (2)
C4—C3—C7	122.5 (2)	C20—C21—C24	118.0 (2)
O2—C4—C8	107.7 (2)	C24—C21—C22	119.2 (2)
C3—C4—O2	122.4 (2)	C18—C22—C21	109.6 (2)
C3—C4—C8	129.8 (3)	C18—C22—H22	108.4
N1—C5—O2	110.2 (2)	C18—C22—C25	111.6 (2)
N1—C5—C6	128.2 (3)	C21—C22—H22	108.4
C6—C5—O2	121.6 (2)	C21—C22—C25	110.2 (2)
C5—C6—C7	123.1 (2)	C25—C22—H22	108.4
C5—C6—C9	118.1 (2)	C19—C23—H23A	109.5
C9—C6—C7	118.8 (2)	C19—C23—H23B	109.5
C3—C7—C6	110.0 (2)	C19—C23—H23C	109.5
C3—C7—H7	108.2	H23A—C23—H23B	109.5
C3—C7—C10	111.6 (2)	H23A—C23—H23C	109.5
C6—C7—H7	108.2	H23B—C23—H23C	109.5
C6—C7—C10	110.6 (2)	N3—C24—C21	179.3 (4)
C10—C7—H7	108.2	C26—C25—C22	121.0 (3)
C4—C8—H8A	109.5	C26—C25—C30	116.4 (3)
C4—C8—H8B	109.5	C30—C25—C22	122.5 (3)
C4—C8—H8C	109.5	F2—C26—C25	118.3 (3)
H8A—C8—H8B	109.5	F2—C26—C27	118.3 (3)
H8A—C8—H8C	109.5	C27—C26—C25	123.4 (4)
H8B—C8—H8C	109.5	C26—C27—H27	120.6
N2—C9—C6	179.1 (4)	C28—C27—C26	118.8 (4)
C11—C10—C7	121.1 (2)	C28—C27—H27	120.6
C11—C10—C15	116.2 (3)	C27—C28—H28	119.7
C15—C10—C7	122.6 (2)	C27—C28—C29	120.5 (4)
F1—C11—C10	117.9 (3)	C29—C28—H28	119.7
F1—C11—C12	118.5 (3)	C28—C29—H29	120.4
C12—C11—C10	123.6 (3)	C28—C29—C30	119.2 (4)
C11—C12—H12	120.4	C30—C29—H29	120.4
C13—C12—C11	119.2 (4)	C25—C30—C29	121.7 (3)
C13—C12—H12	120.4	C25—C30—H30	119.1
C12—C13—H13	120.1	C29—C30—H30	119.1
C12—C13—C14	119.9 (3)	Cl2—C1A—Cl3	104.0 (8)
C14—C13—H13	120.1	Cl2—C1A—H1AA	111.0
C13—C14—H14	120.0	Cl2—C1A—H1AB	111.0
C13—C14—C15	120.0 (4)	Cl3—C1A—H1AA	111.0
C15—C14—H14	120.0	Cl3—C1A—H1AB	111.0
C10—C15—C14	121.2 (3)	H1AA—C1A—H1AB	109.0
C10—C15—H15	119.4	Cl3A—C1B—H1BA	105.6
C14—C15—H15	119.4	Cl3A—C1B—H1BB	105.6
C20—O4—C19	120.4 (2)	H1BA—C1B—H1BB	106.1
H4A—N4—H4B	120.0	Cl2A—C1B—Cl3A	126.8 (17)
C20—N4—H4A	120.0	Cl2A—C1B—H1BA	105.6
C20—N4—H4B	120.0	Cl2A—C1B—H1BB	105.6
H16A—C16—H16B	109.5	Cl1—C1C—H1CA	111.8
H16A—C16—H16C	109.5	Cl1—C1C—H1CB	111.8
H16B—C16—H16C	109.5	Cl4—C1C—Cl1	99.6 (7)
C17—C16—H16A	109.5	Cl4—C1C—H1CA	111.8
C17—C16—H16B	109.5	Cl4—C1C—H1CB	111.8
C17—C16—H16C	109.5	H1CA—C1C—H1CB	109.6
			
F1—C11—C12—C13	178.8 (3)	O4—C20—C21—C22	6.5 (4)
O1—C2—C3—C4	28.1 (4)	O4—C20—C21—C24	−177.1 (2)
O1—C2—C3—C7	−153.5 (3)	N4—C20—C21—C22	−174.2 (3)
O2—C5—C6—C7	0.5 (5)	N4—C20—C21—C24	2.2 (4)
O2—C5—C6—C9	179.9 (3)	C16—C17—C18—C19	−172.5 (3)
N1—C5—C6—C7	−180.0 (3)	C16—C17—C18—C22	7.9 (5)
N1—C5—C6—C9	−0.5 (5)	C17—C18—C19—O4	179.0 (3)
C1—C2—C3—C4	−151.8 (3)	C17—C18—C19—C23	−1.5 (5)
C1—C2—C3—C7	26.7 (4)	C17—C18—C22—C21	−170.5 (3)
C2—C3—C4—O2	179.4 (3)	C17—C18—C22—C25	67.1 (3)
C2—C3—C4—C8	1.9 (5)	C18—C22—C25—C26	−140.8 (3)
C2—C3—C7—C6	179.3 (2)	C18—C22—C25—C30	39.8 (3)
C2—C3—C7—C10	56.2 (3)	C19—O4—C20—N4	−175.8 (2)
C3—C7—C10—C11	−142.6 (2)	C19—O4—C20—C21	3.6 (4)
C3—C7—C10—C15	37.0 (3)	C19—C18—C22—C21	10.0 (4)
C4—O2—C5—N1	178.3 (3)	C19—C18—C22—C25	−112.4 (3)
C4—O2—C5—C6	−2.1 (4)	C20—O4—C19—C18	−6.2 (4)
C4—C3—C7—C6	−2.3 (4)	C20—O4—C19—C23	174.2 (3)
C4—C3—C7—C10	−125.4 (3)	C20—C21—C22—C18	−12.5 (3)
C5—O2—C4—C3	1.3 (4)	C20—C21—C22—C25	110.7 (3)
C5—O2—C4—C8	179.3 (3)	C21—C22—C25—C26	97.1 (3)
C5—C6—C7—C3	1.6 (4)	C21—C22—C25—C30	−82.3 (3)
C5—C6—C7—C10	125.3 (3)	C22—C18—C19—O4	−1.6 (4)
C6—C7—C10—C11	94.6 (3)	C22—C18—C19—C23	178.0 (3)
C6—C7—C10—C15	−85.8 (3)	C22—C25—C26—F2	1.0 (4)
C7—C3—C4—O2	1.0 (4)	C22—C25—C26—C27	−179.8 (3)
C7—C3—C4—C8	−176.4 (3)	C22—C25—C30—C29	180.0 (3)
C7—C10—C11—F1	0.1 (4)	C24—C21—C22—C18	171.2 (2)
C7—C10—C11—C12	179.0 (3)	C24—C21—C22—C25	−65.6 (3)
C7—C10—C15—C14	−178.3 (3)	C25—C26—C27—C28	0.0 (6)
C9—C6—C7—C3	−177.9 (2)	C26—C25—C30—C29	0.5 (4)
C9—C6—C7—C10	−54.2 (3)	C26—C27—C28—C29	0.2 (6)
C10—C11—C12—C13	−0.2 (5)	C27—C28—C29—C30	0.0 (6)
C11—C10—C15—C14	1.4 (5)	C28—C29—C30—C25	−0.4 (5)
C11—C12—C13—C14	0.2 (6)	C30—C25—C26—F2	−179.5 (3)
C12—C13—C14—C15	0.6 (6)	C30—C25—C26—C27	−0.3 (5)
C13—C14—C15—C10	−1.4 (6)	O3A—C17—C18—C19	−13.1 (6)
C15—C10—C11—F1	−179.5 (3)	O3A—C17—C18—C22	167.4 (5)
C15—C10—C11—C12	−0.6 (4)	O3—C17—C18—C19	27.6 (6)
F2—C26—C27—C28	179.2 (3)	O3—C17—C18—C22	−151.9 (4)

### 5-Acetyl-2-amino-4-(2-fluorophenyl)-6-methyl-4H-pyran-3-carbonitrile dichloromethane hemisolvate Hydrogen-bond geometry (Å, º)

**Table:**

*D*—H···*A*	*D*—H	H···*A*	*D*···*A*	*D*—H···*A*
N1—H1*A*···N3^i^	0.86	2.26	3.066 (4)	156
N1—H1*B*···O3^ii^	0.86	2.03	2.844 (8)	159
N1—H1*B*···O3*A*^ii^	0.86	2.02	2.855 (7)	165
N4—H4*A*···N2^iii^	0.86	2.20	3.016 (4)	157
N4—H4*B*···O1^iv^	0.86	2.06	2.910 (3)	170

Symmetry codes: (i) −*x*+1, *y*−1/2, −*z*+1/2; (ii) −*x*+1, −*y*+1, −*z*+1; (iii) −*x*+1, *y*+1/2, −*z*+1/2; (iv) −*x*+1, −*y*+2, −*z*+1.
